# Supplementary material for: Model-checking ecological state-transition graphs
Source: PLoS Comput Biol. 2022 Jun 6;18(6):e1009657. doi: 10.1371/journal.pcbi.1009657 (PMC9203009; doi:10.1371/journal.pcbi.1009657)
Supplement: S1 Appendix — (PDF) [file pcbi.1009657.s001.pdf]

S1 Appendix   If-then rule modelling

The if-then rule language used throughout the paper is called the language of *Reaction Rules* (RR for short). Its syntax (i.e. how models are written) and semantics (i.e. how models are executed) are hereby summarised in an intuitive yet precise form, the full details being available in [1]. We first provide an *explicit semantics*, that consists of operational rules allowing to enumerate all the reachable states. Then, we provide a *symbolic semantics* that is used in `ecco` to obtain memory efficient and human-readable STGs by grouping states.

S1.1   Syntax: writing models

The RR modelling language involves entities and rules [1, 2]. Entities are the biotic and abiotic elements of an ecosystem, modelled as Boolean variables (`on`/`off`, noted as `+`/`-`). Rules define how entities values may evolve by applying an effect (assignment of entities values) depending on a guard (condition on entities values).

Fig S1.1 shows a toy version of our Borana model. Entities are declared first by giving each of them a name, an initial state ("`+`" for `on`, "`-`" for `off`, or "`*`" to allow both initial values), and a textual description. For instance, we declare entity `Gr` that is initially `on` and models the presence of grasses in the ecosystem. With respect to the version presented in the main text, we have added a control `Fb` that has an undefined initial state, so both `Fb+` and `Fb-` are considered as initial states of the system. Entities declarations are organised into arbitrarily chosen categories (except for "`rules`" that is a reserved keyword). For instance, entity `Gr` is declared within category `variables` while entity `Fb` is declared within category `controls`. These categories are for information purposes only and have no consequence on the semantics.

Rules are listed after entities declarations and consist of two sides separated by "`>>`": the left-hand side is the guard, that is, the condition for the execution of the rule; the right-hand side is the effect, that is, the entities assignment that takes place upon execution of the rule. For instance, rule `R1` specifies that if fire is not banned (`Fb-`) and grasses are present (`Gr+`) then high fire may occur, resulting in the disappearance of both shrubs and trees (`Sh-` and `Tr-`). Rules may be prefixed by arbitrary tags enclosed into square brackets, like "`[high fire]`" in rule `R1`. Just like comments, like "`# R1`", these tags play no role in the semantics, but contrasting with comments, they are attached to rules and are available from the tool so they can be referred to during analysis.

```
variables:
  Gr+: Grasses
  Sh-: Shrubs
  Tr-: Trees
controls:
  Fb*: Fire ban
rules:
  [high fire]                Fb-, Gr+ >> Sh-, Tr-      # R1
  [low fire]                 Fb-, Gr+ >> Sh-           # R2
  [browsing]                 Sh+ >> Sh-                # R3
  [grazing + recruitement]   Gr+ >> Sh+, Tr+           # R4
```

Fig S1.1. A toy model of the Borana vegetation. Rules are named for reference using a comment at the end of lines.

Note that Tab 1 and S1 Table have been formatted to be more readable than the raw RR source code, but the latter is available as file "`Borana_model.rr`" in S1 Notebook.

S1.2 Explicit semantics: executing rules and building STGs

The execution of an RR system is defined in terms of operational rules involving *states*, i.e. valuation of its entities, and *transitions*, that are executions (called *firings*) of the rules allowing to build new states from existing ones:

- the *initial states* are defined from the declaration of the entities, either they are initially on/off, or both values are considered (like `Fb*` in Fig S1.1);
- a rule is *enabled* when its guard is satisfied by the state and its effect is not already realised;
- if no rule is enabled at a state, then this state is a *deadlock*;
- firing a rule r enabled at a state s is made by applying the effect of r onto s, yielding a new state $s' \neq s$, which is a transition noted by $s \xrightarrow{r} s'$;
- the states obtained by firing rules from a given state are called its *successors*.

If we note a state by the set of entities it valuates to on, the model defined in Fig S1.1 has two initial states: $\{\texttt{Fb}, \texttt{Gr}\}$ and $\{\texttt{Gr}\}$. Rule `R4` is enabled in both states because we have `Gr+` (condition) but not `Sh+` nor `Tr+` (effect). So it may fire and we have two transitions $\{\texttt{Fb}, \texttt{Gr}\} \xrightarrow{\texttt{R4}} \{\texttt{Fb}, \texttt{Gr}, \texttt{Sh}, \texttt{Tr}\}$ and $\{\texttt{Gr}\} \xrightarrow{\texttt{R4}} \{\texttt{Gr}, \texttt{Sh}, \texttt{Tr}\}$. In the initial states, rule `R3` is not enabled because its guard is not satisfied. Rules `R1` and `R2` are not enabled from the initial states either because in $\{\texttt{Fb}, \texttt{Gr}\}$, even is the guard is satisfied, the effect is already realised, and in $\{\texttt{Gr}\}$ the guard is not satisfied.

An STG can be generated from an RR model by repeatedly applying the firing rules from the initial states and from all the newly obtained successor states. The STG obtained from our toy model is depicted in Fig S1.2 and is computed as follows:

- we start from the initial states $\{\texttt{Fb}, \texttt{Gr}\}$ and $\{\texttt{Gr}\}$ (drawn at the top);
- the possible transitions are:
  - $\{\texttt{Fb}, \texttt{Gr}\} \xrightarrow{\texttt{R4}} \{\texttt{Fb}, \texttt{Gr}, \texttt{Sh}, \texttt{Tr}\}$,
  - $\{\texttt{Gr}\} \xrightarrow{\texttt{R4}} \{\texttt{Gr}, \texttt{Sh}, \texttt{Tr}\}$,

  which yield two new states (drawn in the middle);
- then, the possible transitions are:
  - $\{\texttt{Fb}, \texttt{Gr}, \texttt{Sh}, \texttt{Tr}\} \xrightarrow{\texttt{R3}} \{\texttt{Fb}, \texttt{Gr}, \texttt{Tr}\}$,
  - $\{\texttt{Gr}, \texttt{Sh}, \texttt{Tr}\} \xrightarrow{\texttt{R1}} \{\texttt{Gr}\}$,
  - $\{\texttt{Gr}, \texttt{Sh}, \texttt{Tr}\} \xrightarrow{\texttt{R2}} \{\texttt{Gr}, \texttt{Tr}\}$,
  - $\{\texttt{Gr}, \texttt{Sh}, \texttt{Tr}\} \xrightarrow{\texttt{R3}} \{\texttt{Gr}, \texttt{Tr}\}$,

  which yield only two new states (drawn at the bottom), one of which being obtained twice, and the last transition yielding a state we already had;
- then, the possible transitions are:
  - $\{\texttt{Fb}, \texttt{Gr}, \texttt{Tr}\} \xrightarrow{\texttt{R4}} \{\texttt{Fb}, \texttt{Gr}, \texttt{Sh}, \texttt{Tr}\}$,
  - $\{\texttt{Gr}, \texttt{Tr}\} \xrightarrow{\texttt{R1}} \{\texttt{Gr}\}$,

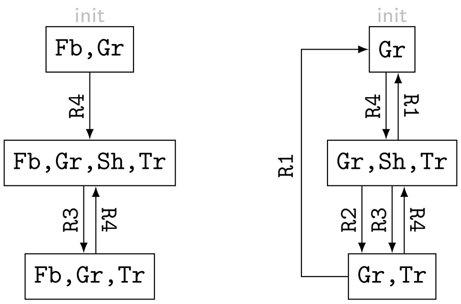

Fig S1.2. The STG generated from the toy RR model of Fig S1.1.

- $\{\mathtt{Gr}, \mathtt{Tr}\} \xrightarrow{\mathtt{R4}} \{\mathtt{Gr}, \mathtt{Sh}, \mathtt{Tr}\}$,

which do not yield any new state, so the computation stops.

The STG resulting from this computation exhibits two disjoint subgraphs corresponding to the two initial values of control `Fb`. The right-most subgraph is exactly that of Fig 2.

S1.3 Symbolic semantics: building smaller STGs

Symbolic techniques efficiently build a compact representation of the set S of states of an RR model. Then, considering a partition C of S, we can build an STG whose nodes are the elements of C and whose edges are the transitions allowing to reach one such node from another. More precisely, for $s \in S$, we note by $\langle s \rangle_C$ the component in C such that $s \in \langle s \rangle_C$. Then, the symbolic STG of an RR system with respect to C is the STG defined by:

$$\left(C \quad , \quad \{ (\langle s \rangle_C, r, \langle s' \rangle_C) \mid s \xrightarrow{r} s' \wedge \langle s \rangle_C \neq \langle s' \rangle_C \} \right).$$

For example, the STG depicted in blue in Fig 6 is a symbolic STG whose components are the blue rounded boxes, each representing a vegetation class (indeed the vegetation classes form a partition, see S2 Table) and encompassing the explicit states it encloses. To draw this STG, we first symbolically computed the symbolic STG before enumerating the states enclosed within each component (see S1 Notebook).

The main benefit of such symbolic STG is that its components may be represented symbolically, and that its edges can be computed directly from the symbolic components. In doing so, a huge state-space may be represented efficiently by a small structure that can be seen as a hybrid object mixing symbolic components with explicit information about their relationship.

The simplest symbolic STG has a single component ($C = \{S\}$), and the most detailed symbolic STG has only singleton components ($C = \{\{s\} \mid s \in S\}$) which is equivalent to the explicit STG as defined above. Yet, we are mostly interested in something in between with sufficiently few components to remain human-readable, but with enough details to exhibit key aspects of the dynamics.

References

1. Pommereau F, Thomas C, Gaucherel C. Petri nets semantics of Reaction Rules (RR). In: Proc. of PETRINETS'22. vol. to appear of LNCS. Springer; 2022.

2. Gaucherel C, Pommereau F. Using discrete systems to exhaustively characterize the dynamics of an integrated ecosystem. Methods in Ecology and Evolution. 2019;10(9):1615–1627. doi:10.1111/2041-210X.13242.
