# Supplementary material for: Model-checking ecological state-transition graphs
Source: PLoS Comput Biol. 2022 Jun 6;18(6):e1009657. doi: 10.1371/journal.pcbi.1009657 (PMC9203009; doi:10.1371/journal.pcbi.1009657)
Supplement: S2 Appendix — (PDF) [file pcbi.1009657.s002.pdf]

S2 Appendix Justification of the *Borana model*

S2.1 Modelling methodology

The *Borana model* is based on studies about the vegetation dynamics of the Borana Zone in Ethiopia [1–4].

> *"The complexity of rangeland vegetation dynamics can be interpreted by the state-and-transition model, in which rangeland dynamics are described as a set of discrete "states" of vegetation at a specific site and changes between states that occur as discrete "transitions"." [2, p.2]*

> *"Transitions from one state to another often require a combination of climatic circumstances and management actions (e.g., fire or grazing) to bring them about." [4, p.7]*

The *Borana model* represents the discrete vegetation states by a set of Boolean variables, other Boolean variables called controls also cover management actions and climatic circumstances. The transitions between states are described by *if-then* rules linking a *condition* (on the values of the variables) with a *consequence* (an update of the variables).

> *"Therefore, spatial knowledge of current vegetation states plus understanding of past and future transition pathways is needed to properly prescribe and apply efforts to mitigate undesirable processes such as bush encroachment. The goal of this study was to provide pastoralists, rangeland managers, and policy makers with a spatial understanding of the past, current, and potential rangeland vegetation states in Borana" [2, p.2]*

In order to foresee the future transition pathways, the *Borana model* is not limited to a description of the observed transition pathways (the STGs available in [2–4]). From a set of initial states, the *Borana model* computes every state reachable by the cascading applications of if-then rules. Thus the *Borana model* outputs unobserved transition pathways, assuming that the vegetation dynamics can be deduced from the description of the discrete transitions (i.e. the set of if-then rules). In consequence, this methodology can be used to foresee the effects of new ecosystem management policies, as long as they result into discrete transitions.

S2.2 Variables

We chose five vegetation variables ($\texttt{Gr}, \texttt{Sh}, \texttt{Tr}, \texttt{Sa}, \texttt{Cr}$, see Tab 1 left panel) to represent the eight vegetation classes (S2 Table) forming the states of the Borana STMs [2]. We then added three variables representing the presence of grazers or browsers ($\texttt{Lv}, \texttt{Gz}, \texttt{Bw}$, see Tab 1 left panel).

> *"While climatic and edaphic factors primarily determine broad-scale vegetation distribution, complex patches of open and closed canopy rangelands can exist within a single climate zone, suggesting that controls such as fire and herbivory are important at a finer spatial scale." [2, p.3]*

> *"Mean annual rainfall ranges from* $300mm$ *in the lowlands to* $1,000mm$ *in the highlands. (...) Generally, annual precipitation is positively correlated with elevation." [2, p.2]*

> *"The government prohibited grazing in such forested areas for conservation purposes" [2, p.8]*

> *"Until the 1950s, crop cultivation throughout the Borana Zone was banned by indigenous rules (...) In recent years, commercial farming has become more prevalent." [2, p.8]*

> *"Adding more goats and camels while reducing the number of cattle in the herds could be crucial (...) Rather than simply living with bush encroachment, pastoralists can actively contribute to its mitigation by changing their livestock portfolios." [2, p.8-9]*

We defined seven control variables (Tab 1 right panel), representing climate/altitude (`Alt`), fire ban (`Fb`), crop ban (`Cb`), or herbivory (`Wl`, `Ps`, `Ig`, `BLv`). Controls influence the system but cannot change along the dynamics (S1 Table), thus each valuation of the controls represents a specific scenario.

S2.3 Initial states

> *"Since the 1970s, fire has been banned, leaving livestock grazing as major local-level disturbance factor. The last high-intensity fire set the rangeland state as grassland." [3, p.7]*

The initial states represent the grassland vegetation class (grasses are the only present vegetation) after the last high-intensity fire (no animals). Hence the only variable initially valuated `+` is `Gr` (Tab 1 left panel). There is a single initial state per scenario (i.e. control valuation), thus there are $2^7 = 128$ initial states.

S2.4 Rules

Fire rules: `R1, R2`

> *"Since the 1970s, fire has been banned." [3, p.7]*

Hence `Fb-` in the condition of `R1, R2`.

> *"Low intensity fires would periodically burn grasses, shrubs, and tree saplings on sparse scrubland, but would leave adult trees undamaged." [3, p.7]*

Hence `Sh-, Sa-` in the consequence of `R1`.

> *"High intensity but low frequency fire could change the landscape into a grass-dominated system." [3, p.7]*

Hence `Sh-, Tr-, Sa-` in the consequence of `R2`.

> *"Although bush burning ban has been lifted since the 2000s, herbaceous biomass in the understory was minimal, and fuel loads would not build up and could not set the stage for fires to properly thin the woody layer." [3, p.7]*

Hence `Gr+` in the condition of `R1, R2`.

> *"Grasses and other herbs usually established themselves first after fire" [3, p.7]*

Hence we did not set `Gr-` in the consequence of `R1` and `R2`. Thus, as `Gr+` is in the condition of `R1` and `R2`, it is still present after their application.

Finally, animals are not mentioned during fire descriptions [3,4], we assumed that they flee. Hence `Lv-, Gz-, Bw-` in the consequence of `R1` and `R2`.

Trees recruitment: `R3`

> *"Given favorable environmental conditions, tree seedlings could grow into mature trees and gradually close the canopy." [1, p.43]*

Grass recruitment: `R4`

> *"Grasses and other herbs usually established themselves first after fire" [3, p.7]*

We assumed that grasses established themselves first after any perturbation clearing the vegetation cover.

Closed Canopy Woodland transition: `R5`

> *"In the highlands (...) given higher precipitation and absence of fire, tree seedlings in dense scrubland could grow into mature trees and gradually close the canopy." [2, p.8]*

Hence `Alt+` (*"in the highlands"*), `Fb+` (*"absence of fire"*), and `Gr-` (*"in dense scrubland"*) in condition of `R3`.

Bushland transition: `R6`

> *"(Bushland) at the higher end of the (elevation) range is shifting into dense scrubland. (...) In the relatively dry lowlands of the Borana Zone, the primary vegetation transition is from grassland to bushland." [2, p.8]*

We chose to represent this fact by the competitive exclusion of tree saplings by shrubs at low altitude `Alt-`.

Grazers: `R7`

We assumed that the presence of wild grazers `Gz+` is conditioned by the presence of grasses `Gr+` and by the absence of livestock `Lv-`.

Browsers: `R8, R9`

We assumed that the presence of wild browsers `Bw+` is conditioned by the presence of shrubs `Sh+` (`R8`) or saplings `Sa+` (`R9`), and by the absence of livestock `Lv-`.

Livestock: `R10, R11, R12`

We assumed that the presence of livestock `Lv+` is conditioned by the presence of grasses `Gr+` (`R10`), or under browsing livestock policies `BLv+` by the presence of shrubs `Sh+` (`R11`) or saplings `Sa+` (`R12`). We also assumed that livestock `Lv` excludes both wild grazers `Gz` and wild browsers `Bw`.

Grazing: `R13, R14`

> *"With woody plant recruitment and wildlife grazing, the grassland could gradually shift into a sparse scrubland state. (...) Light to moderate grazing reduced understory cover of grassland, and the system shifted into sparse scrubland given plant recruitment.(...) Similarly, on the open canopy woodland, woody plants would also become denser given moderate grazing pressure, gradually shifting the rangeland into the sparse scrubland state." [3, p.7]*

Intensive grazing: `R15`

> *"Heavy grazing on the sparse scrubland could diminish forage in the understory within a short time period, thus leaving the scattered woody plants free from competition." [3, p.7]*

> *"As pastoralists sedentarize and herd livestock near and around their settlements in response to external sedentarization initiatives, rangelands can shift into bare ground or shrublands with minimal grazing value." [4, p.2]*

Browsing: `R16, R17`

> *"Wildlife browsing could keep the re-sprouting woody species in check." [3, p.7]*

> *"Increasing browsing pressure by goats and camels can thin the woody plant layer and suppress the growth of shrubs and trees, which can indirectly facilitate the growth of herbs on the ground." [2, p.8]*

We chose to represent this fact by enabling browsers (wild `Bw` or domestic `BLv` $\wedge$ `Lv`) to remove shrubs `Sh` and saplings `Sa`. Grasses `Gr` are supposed to establish themselves first in the cleared space.

Crops: `R18, R19`

> *"Dense scrublands, along with other minor classes such as closed and open canopy woodlands, that are situated at above* $1,200m$ *are being converted to cultivated areas, which allows the practice of rain-fed agriculture." [2, p.8]*

> *"Cropland expansion (...) accelerate rangeland degradation and wildlife habitat loss, and discourage mobile livestock herding" [4, p.2]*

We chose to represent dense scrublands, closed and open canopy woodland by the presence of trees (see S2 Table). Hence the condition of `R18`: high altitude `Alt+`, crops being allowed `Cb-` and trees `Tr+`. The consequence of `18` includes the replacement of grasses `Gr`, shrubs `Sh` and saplings `Sa` by cultivated species `Cr`, and the disappearance of both livestock `Lv` and wild life `Gz, Bw`.

> *"During the 2003–2013 decade,* $355km^2$ *of cropland transitioned backed to dense scrublands and* $124km^2$ *back to open canopy woodlands." [2, p.7]*

We assumed grasses establish themselves first after crops are abandoned (`R19`).

S2.5 Improvements

Here we list some worth considering improvements, unfortunately unavailable data would be required in order to implement them:

- Add a variable representing bare soil. Indeed the definition of the vegetation classes [2, Tab.1] is based on vegetation cover, which does not always sum up to 100%. Thus the percentage of bare soil is a part of the vegetation classes description. Nevertheless, bare soil is almost never mentioned in the sources, so we chose to not include it in the variables.
- Represent species instead of plant functional types. Indeed the vegetation classes [2, Tab.1] do not encompass the same species of grasses, shrubs nor trees. Unfortunately, data on the species dynamics is lacking for the Borana zone.

- Desynchronise shrubs and saplings browsing. The browsing rules (`R16, R17`) remove saplings and shrubs simultaneously, but it may not be the case in reality (because of food preference for example). Without more precise data, we chose to synchronise both removals.

- Remove the scenarios including pastoralism policies (`Ig+` or `BLv+`) but without pastoralism (`Ps-`). In consequence, the STG would be limited to $2^7 - 3 \times 2^4 = 80$ disconnected scenarios: one for each of the 2^7 combinations of the controls valuations, minus the 3×2^4 valuations including pastoralism policies (`Ig+` or `BLv+` or both) but without pastoralism (`Ps-`). We did not remove those scenarios in order to ease the presentation of the *Borana model*. Note that those scenarios are only duplicates of the scenarios without pastoralism policies at all (`Ps-, Ig-, BLv-`), and thus they do not introduce unwanted behaviours but only enlarge the STG.

References

1. Liao C. Complexity In The Open Grazing System: Rangeland Ecology, Pastoral Mobility And Ethnobotanical Knowledge In Borana, Ethiopia [PhD Thesis]. Cornell University; 2016. Available from: https://hdl.handle.net/1813/43578.

2. Liao C, Clark PE, DeGloria SD. Bush encroachment dynamics and rangeland management implications in southern Ethiopia. Ecology and Evolution. 2018;8(23):11694–11703. doi:10.1002/ece3.4621.

3. Liao C, Clark PE. Rangeland vegetation diversity and transition pathways under indigenous pastoralist management regimes in southern Ethiopia. Agriculture, Ecosystems & Environment. 2018;252:105–113. doi:10.1016/j.agee.2017.10.009.

4. Liao C, Agrawal A, Clark PE, Levin SA, Rubenstein DI. Landscape sustainability science in the drylands: mobility, rangelands and livelihoods. Landscape Ecology. 2020;35(11):2433–2447. doi:10.1007/s10980-020-01068-8.
