# Supplementary material for: Model-checking ecological state-transition graphs
Source: PLoS Comput Biol. 2022 Jun 6;18(6):e1009657. doi: 10.1371/journal.pcbi.1009657 (PMC9203009; doi:10.1371/journal.pcbi.1009657)
Supplement: S1 Table — The 19 if-then rules describing the vegetation dynamics in Borana. (PDF) [file pcbi.1009657.s003.pdf]

S1 Table Ruleset of the *Borana model*

Table S1. Ruleset of the *Borana model*. The 19 if-then rules describing the vegetation dynamics in Borana. The left part of a rule is its condition (if ...), and the right part is its consequence (then ...). For example, the first rule R1 expresses that *if* fire is allowed (Fb-) and grasses are present (Gr+), *then* (>>) a low intensity fire can occur, resulting in the burning of shrubs and saplings (Sh-, Sa-) and in the animals escape (Lv-, Gz-, Bw-), as grasses resprout first they do not disappear in the fire consequence. This ruleset is inspired from the STM literature on Borana [1–4], see S2 Appendix.

	Description	Condition (if ...)		Consequence (then ...)
R1	*Low fire*	Fb-, Gr+	>>	Sh-, Sa-, Lv-, Gz-, Bw-
R2	*High fire*	Fb-, Gr+	>>	Sh-, Tr-, Sa-, Lv-, Gz-, Bw-
R3	*Trees*	Sa+	>>	Tr+
R4	*Grass*	Sh-, Tr-, Sa-, Cr-	>>	Gr+
R5	*CCW*	Alt+, Fb+, Gr-, Sa+	>>	Sh-, Tr+
R6	*Bushland*	Alt-, Sh+, Tr-	>>	Sa-
R7	*Grazers*	Wl+, Gr+, Lv-	>>	Gz+
R8	*Browsers*	Wl+, Sh+, Lv-	>>	Bw+
R9	*Browsers*	Wl+, Sa+, Lv-	>>	Bw+
R10	*Livestock*	Ps+, Gr+	>>	Lv+, Gz-, Bw-
R11	*Livestock*	Ps+, BLv+, Sh+	>>	Lv+, Gz-, Bw-
R12	*Livestock*	Ps+, BLv+, Sa+	>>	Lv+, Gz-, Bw-
R13	*Grazing*	Gr+, Lv+	>>	Sh+, Sa+
R14	*Grazing*	Gr+, Gz+	>>	Sh+, Sa+
R15	*Intensive grazing*	Ig+, Lv+	>>	Gr-, Lv-
R16	*Browsing*	Bw+	>>	Gr+, Sh-, Sa-, Bw-
R17	*Browsing*	BLv+, Lv+	>>	Gr+, Sh-, Sa-, Bw-
R18	*Crops*	Alt+, Cb-, Tr+	>>	Gr-, Sh-, Sa-, Cr+, Lv-, Gz-, Bw-
R19	*Crops*	Cr+	>>	Gr+, Cr-

References

1. Liao C. Complexity In The Open Grazing System: Rangeland Ecology, Pastoral Mobility And Ethnobotanical Knowledge In Borana, Ethiopia [PhD Thesis]. Cornell University; 2016. Available from: https://hdl.handle.net/1813/43578.

2. Liao C, Clark PE. Rangeland vegetation diversity and transition pathways under indigenous pastoralist management regimes in southern Ethiopia. Agriculture, Ecosystems & Environment. 2018;252:105–113. doi:10.1016/j.agee.2017.10.009.

3. Liao C, Clark PE, DeGloria SD. Bush encroachment dynamics and rangeland management implications in southern Ethiopia. Ecology and Evolution. 2018;8(23):11694–11703. doi:10.1002/ece3.4621.

4. Liao C, Agrawal A, Clark PE, Levin SA, Rubenstein DI. Landscape sustainability science in the drylands: mobility, rangelands and livelihoods. Landscape Ecology. 2020;35(11):2433–2447. doi:10.1007/s10980-020-01068-8.
