# Supplementary material for: Model-checking ecological state-transition graphs
Source: PLoS Comput Biol. 2022 Jun 6;18(6):e1009657. doi: 10.1371/journal.pcbi.1009657 (PMC9203009; doi:10.1371/journal.pcbi.1009657)
Supplement: S2 Table — Borana vegetation classes [22] translated into state properties (presence or absence of vegetation variables). (PDF) [file pcbi.1009657.s004.pdf]

S2 Table Borana vegetation classes as state properties

Table S2. Borana vegetation classes as state properties. Borana vegetation classes [1] translated into state properties (presence or absence of vegetation variables). A variable always present in the vegetation class is noted with "+", a variable always absent is noted with "-", while a variable whose presence is variable in the vegetation class is noted with "*". For example, closed canopy woodland is described by the state property stating that trees are present, grasses, shrubs and crops are absent, while saplings can be either present or absent. Note that grasses are considered functionally present in the sparse scrubland class although covering only between 10% and 30% of the surface [1], indeed both fire and grazing occur in sparse scrublands [2].

Vegetation Class	State property
Closed Canopy Woodland	Gr-, Sh-, Tr+, Sa*, Cr-
Dense Scrubland	Gr-, Sh+, Tr+, Sa*, Cr-
Bushland	Gr-, Sh+, Tr-, Sa*, Cr-
Open Canopy Woodland	Gr+, Sh-, Tr+, Sa*, Cr-
Sparse Scrubland	Gr+, Sh+, Tr*, Sa*, Cr-
Cultivated Land	Gr-, Sh-, Tr*, Sa-, Cr+
Grassland	Gr+, Sh-, Tr-, Sa-, Cr-
Sparsely Vegetated Land	Gr-, Sh-, Tr-, Sa-, Cr-

References

1. Liao C, Clark PE, DeGloria SD. Bush encroachment dynamics and rangeland management implications in southern Ethiopia. Ecology and Evolution. 2018;8(23):11694–11703. doi:10.1002/ece3.4621.

2. Liao C, Agrawal A, Clark PE, Levin SA, Rubenstein DI. Landscape sustainability science in the drylands: mobility, rangelands and livelihoods. Landscape Ecology. 2020;35(11):2433–2447. doi:10.1007/s10980-020-01068-8.
