# Supplementary material for: Model-checking ecological state-transition graphs
Source: PLoS Comput Biol. 2022 Jun 6;18(6):e1009657. doi: 10.1371/journal.pcbi.1009657 (PMC9203009; doi:10.1371/journal.pcbi.1009657)
Supplement: S1 Notebook — Zip archive containing: (1) “README” is a text file explaining how to install ecco (Section 1.2.2), (2) “Borana_model.rr” is a text file containing the system description of the Borana model (Table 1 and S1 Table), (3) “S1_notebook.ipynb” is a Jupyter notebook covering the Borana model analysis (Section 2), (4) “S1_notebook.html” is a static HTML preview of this notebook. (ZIP) [file pcbi.1009657.s005.zip › S1_Notebook/S1_notebook.html]

S1\_notebook


# Model-checking ecological state-transition graphs (S1 Notebook)¶

## Borana vegetation pathways model¶

Computation of the STG.

In [1]:

```
# print the execution time
%time
# compute the Borana model 
%run -m ecco Borana_model.rr

controls = ["Alt", "Fb", "Cb", "Wl", "Ps", "Ig", "BLv"]
# build the STG
G = ComponentGraph.from_model(model, compact=False, split=False)
# split the initial states (one per scenarios)
v = G.split("INIT")
# print the model statistics
print(f"The STG has {sum ([len(c) for c in G])} states encompassing {sum ([len(v[i]) for i in v.isin('INIT')])} scenarios.")

v = G.split(*controls)
print(f"The largest scenario subgraph has {max([len(c) for c in v])} states.")
```

```
CPU times: user 2 µs, sys: 0 ns, total: 2 µs
Wall time: 5.48 µs
```

```
The STG has 1185 states encompassing 128 scenarios.
The largest scenario subgraph has 26 states.
```

The system description of the Borana model (variables and ruleset, corresponding to Tab 1 and S1 Table):

In [2]:

```
model.rr
```

## Comparison with Liao's STMs¶

Based on the vegetation classes of (Liao et al. 2018a, Tab. 1) we defined vegetation classes as state properties (presence and absence of vegetation variables), see S2 Table. The variables noted with "+" are present, those with "-" are absent, while those with "\*" can be either present or absent.

| Vegetation Class | State Description |
| --- | --- |
| Closed Canopy Woodland | $\texttt{Gr-, Sh-, Tr+, Sa\*, Cr-}$ |
| Dense Scrubland | $\texttt{Gr-, Sh+, Tr+, Sa\*, Cr-}$ |
| Bushland | $\texttt{Gr-, Sh+, Tr-, Sa\*, Cr-}$ |
| Open Canopy Woodland | $\texttt{Gr+, Sh-, Tr+, Sa\*, Cr-}$ |
| Sparse Scrubland | $\texttt{Gr+, Sh+, Tr\*, Sa\*, Cr-}$ |
| Cultivated Land | $\texttt{Gr-, Sh-, Tr\*, Sa-, Cr+}$ |
| Grassland | $\texttt{Gr+, Sh-, Tr-, Sa-, Cr-}$ |
| Sparsely Vegetated Land | $\texttt{Gr-, Sh-, Tr-, Sa-, Cr-}$ |

In [3]:

```
VegetationClass = {}
VegetationClass["ClosedCanopyWoodland"] = "(~Gr & ~Sh & Tr & ~Cr)"
VegetationClass["DenseScrubland"] = "(~Gr & Sh & Tr & ~Cr)"
VegetationClass["Bushland"] = "(~Gr & Sh & ~Tr & ~Cr)"
VegetationClass["OpenCanopyWoodland"] = "(Gr & ~Sh & Tr & ~Cr)"
VegetationClass["SparseScrubland"] = "(Gr & Sh & ~Cr)"
VegetationClass["Cropland"] = "(~Gr & ~Sh & ~Sa & Cr)"
VegetationClass["Grassland"] = "(Gr & ~Sh & ~Tr & ~Sa & ~Cr)"
VegetationClass["SparselyVegetatedLand"] = "(~(Gr | Sh | Tr | Sa | Cr))"
```

In order to compare various scenarios our Borana model with Liao's STMs (Liao et al. 2018b, Fig. 5), we split the states of the Borana model between the vegetation classes. Thus the STGs drawn below aggregate the states of our Borana model into vegetation classes. An edge is drawn between two vegetation classes if and only if there is at least one transition in the STG of the model from a state of the first vegetation class to a state of the second vegetation class. Those edges are labelled with the corresponding rules of the model's STG.

Nodes (vegetation classes) decorated with a downward triangle contain initial states, nodes decorated with a circle contain cyclic pathways. The nodes colors are chosen arbitrarily.

### Computation of Fig 6¶

The blue rounded boxes of Fig 6 gathering the states within the same vegetation classes are displayed here as node colors, i.e. the nodes with the same color are within the same box.

In [4]:

```
# compute the STG with Pastoralism, Cropban and without Fireban, Intensive Grazing, Wildlife and Browsing Livestock
g = ComponentGraph.from_model(model, compact=False, init="Alt+,Fb-,Cb+,Wl+,Ps-,Ig-,BLv-", split=False)
# split into vegetation classes
v = g.split(**VegetationClass)
v = v.explicit()
v.n["lbl"] = lambda n : f"{n.ISIN} / {n.on - set(controls)}"
# draw the graph
v.draw(fig_width=600, fig_height=400, nodes_label="lbl", nodes_color="component", fig_padding=0.15)
```

### Before livestock introduction¶

In [5]:

```
# compute the STG without Fireban, Pastoralism (and related policies), but with Cropban and Wildlife
g = ComponentGraph.from_model(model, compact=False, init="Alt*,Fb-,Cb+,Wl+,Ps-,Ig-,BLv-", split=False)
# split into vegetation classes
v = g.split(**VegetationClass)
# draw the graph
v.draw(fig_width=800, fig_height=250, nodes_label="EQUALS", nodes_color="node", fig_padding=0.1)
```

The STG and the STM of (Liao et al. 2018b, Fig. 5A), figure redone with author's permission, are almost identical:

The only difference is the the additional labels "browsing" and "low fire" on the transition between sparse scrubland and grassland. Indeed those events may happen in sparse scrubland before the establishment of trees.

### With livestock and fire¶

In [6]:

```
# compute the STG with Pastoralism, Cropban and without Fireban, Intensive Grazing, Wildlife and Browsing Livestock
g = ComponentGraph.from_model(model, compact=False, init="Alt*,Fb-,Cb+,Wl-,Ps+,Ig-,BLv-", split=False)
# split into vegetation classes
v = g.split(**VegetationClass)
# draw the graph
v.draw(fig_width=800, fig_height=250, nodes_label="EQUALS", nodes_color="node", fig_padding=0.1)
```

The STG and the STM of (Liao et al. 2018b, Fig. 5B), figure redone with author's permission, are almost identical::

The only difference is the the additional label "low fire" on the transition between sparse scrubland and grassland. Indeed this event may happen in sparse scrubland before the establishment of trees.

### With intensive grazing and fireban¶

In [7]:

```
# compute the STG with Fireban, Intensive Grazing, Cropban, and without Wildlife and Browsing Livestock, starting both from Grassland and Open Canopy Woodland
g = ComponentGraph.from_model(model, compact=False, init="Alt*,Fb+,Cb+,Wl-,Ps+,Ig+,BLv-,Tr*", split=False)
# split into vegetation classes
v = g.split(**VegetationClass)
# draw the graph
v.draw(fig_width=800, fig_height=250, nodes_label="EQUALS", nodes_color="node", fig_padding=0.1)
```

The STG is similar to the STM of (Liao et al. 2018b, Fig. 5C), figure redone with author's permission:

Some of the extra transitions were empirically observed, such as the transition between Dense Scrubland and Closed Canopy Woodland, as well as the extra vegetation classes (Liao et al. 2018a, Tab. 1, Fig. 5), in particular sparse scrubland is described as a transitory state between grassland and dense scrubland. The main features of the STM: (1) that encroachment is not reversible, are encounterd again in the STG, and (2) that open canopy woodland is not reachable from grassland. See also (Liao thesis, 2016, Fig. 6 p31) for a STG representation of (Liao et al. 2018a, Fig. 5).

## Scenario selection by model-checking¶

| English description of the pattern | CTL Formula |
| --- | --- |
| **Reachability pattern** |  |
| An $x$ state *can* be reached | $EF(x)$ |
| An $x$ state *cannot* be reached | $\neg EF(x)$ |
| **Consequence pattern** |  |
| If an $x$ state is reached, then it is *possibly* followed by an $y$ state | $AG(x \Rightarrow EF(y))$ |
| If an $x$ state is reached, then it is *necessarily* followed by an $y$ state | $AG(x \Rightarrow AF(y))$ |
| **Sequence pattern** |  |
| An $y$ state is reachable and is *possibly* preceded *at some time* by an $x$ state | $EF(x \land EF(y))$ |
| An $y$ state is reachable and is *possibly* preceded *all the time* by an $x$ state | $E(x U y)$ |
| An $y$ state is reachable and is *necessarily* preceded *at some time* by an $x$ state | $EF(y) \land \neg E( \neg x U y)$ |
| An $y$ state is reachable and is *necessarily* preceded *all the time* by an $x$ state | $EF(y) \land AG(\neg x \Rightarrow AG(\neg y))$ |
| **Invariance pattern** |  |
| $x$ states *can* persist forever | $EG(x)$ |
| $x$ states *must* persist forever | $AG(x)$ |
| $x$ states *possibly* remain forever reachable | $EG(EF(x))$ |
| $x$ states *necessarily* remain forever reachable | $AG(EF(x))$ |
| $x$ states are *necessarily* reached infinitely often | $AG(AF(x))$ |
| **Reachability & Invariance pattern** |  |
| It is *possible* to reach a state from which $x$ states *can* persist forever | $EF(EG(x))$ |
| It is *possible* to reach a state from which $x$ states *must* persist forever | $EF(AG(x))$ |

The queries are built upon the following state properties :

- Closed canopy woodland is a vegetation class modelled by the presence and absence of plant variables : $$\texttt{ClosedCanopyWoodland} = \texttt{Gr-} \land \texttt{Sh-} \land \texttt{Tr+} \land \texttt{Cr-}$$
- Encroachment is modelled by the vegetation classes with trees or shrubs but without grasses nor crops (closed canopy woodland, dense scrubland and bushland) : $$\texttt{Encroachment} = (\texttt{Tr+} \lor \texttt{Sh+}) \land \texttt{Gr-} \land \texttt{Cr-}$$
- Subsistence production is modelled by the states with livestock or crops : $$\texttt{Subsistence} = \texttt{Lv+} \lor \texttt{Cr+}$$

In [8]:

```
closed_canopy_woodland = "(~Gr & ~Sh & Tr & ~Cr)"
encroachment = "((Tr | Sh) & ~Gr & ~Cr)"
subsistence = "(Lv | Cr)"
```

### Reachability pattern : EF(Encroachment)¶

Pattern : $\exists F (\texttt{Encroachment})$

Pattern description : An encroached state can be reached.

In [9]:

```
%%time
# translation of the pattern into a CTL formula f
f = f"EF({encroachment})"
# split the states whether they satisfy f or not
v = G.split(f)
# split the states whether they are initial or not
v = v.split("INIT")
# print the scenarios satisfying f
v.form(*(v.isin(f) & v.isin("INIT")), variables=controls)
```

```
CPU times: user 967 ms, sys: 7.53 ms, total: 974 ms
Wall time: 974 ms
```

Out[9]:

$\displaystyle Ig \wedge Ps$

Encroachment can only happen under the scenarios encompassing pastoralism $\texttt{Ps+}$ with intensive grazing $\texttt{Ig+}$.

### Reachability pattern : EF(ClosedCanopyWoodland)¶

Pattern : $\exists F (\texttt{ClosedCanopyWoodland})$

Pattern description : Closed Canopy Woodland can be reached.

In [10]:

```
%%time
# translation of the pattern into a CTL formula f
f = f"EF({closed_canopy_woodland})"
# split the states whether they satisfy f or not
v = G.split(f)
# split the states whether they are initial or not
v = v.split("INIT")
# print the scenarios satisfying f
v.form(*(v.isin(f) & v.isin("INIT")), variables=controls)
```

```
CPU times: user 949 ms, sys: 0 ns, total: 949 ms
Wall time: 948 ms
```

Out[10]:

$\displaystyle Ig \wedge Ps \wedge \left(Alt \vee BLv \vee Wl \vee \neg Fb\right)$

Closed Canopy Woodland can only happen under pastoralism $\texttt{Ps+}$ with intensive grazing $\texttt{Ig+}$ and with at least one of the following factors: high altitude $\texttt{Alt+}$, no fire ban $\texttt{Fb-}$, presence of wildlife $\texttt{Wl+}$, browsing livestock $\texttt{BLv+}$.

### Reachability + Consequence pattern : EF(Encroachment) & AG(Encroachment => EF(!Encroachment))¶

Pattern : $\exists F(\texttt{Encroachment}) \land \forall G ( \texttt{Encroachment} => \exists F(\neg \texttt{Encroachment}))$

Pattern description : An encroached state is reachable, and whenever it is reached it is possibly followed by an unencroached state, i.e. it is reversible.

In [11]:

```
%%time
# translation of the pattern into a CTL formula f
f = f"EF({encroachment}) & (AG({encroachment} => EF(~({encroachment}))))"
# split the states whether they satisfy f or not
v = G.split(f)
# split the states whether they are initial or not
v = v.split("INIT")
# print the scenarios satisfying f
v.form(*(v.isin(f) & v.isin("INIT")), variables=controls, normalise="dnf")
```

```
CPU times: user 399 ms, sys: 0 ns, total: 399 ms
Wall time: 398 ms
```

Out[11]:

$\displaystyle Alt \wedge Ig \wedge Ps \wedge \neg Cb$

If an encroached state is reachable ($\texttt{Ps+} \land \texttt{Ig+}$, see query 1) and if the system is at high altitude $\texttt{Alt+}$ with crops allowed $\texttt{Cb-}$, then whenever an encroached state is reached it is possibly followed by an unencroached state.

### Sequence pattern : EF(Encroachment & EF(!Encroachment))¶

Pattern : $\exists F(\texttt{Encroachment} \land \exists F(\neg\texttt{Encroachment}))$

Pattern description : An unencroached state is reachable and is possibly preceded at some time by an encroached state, i.e. at least some encroachment pathways are reversible.

In [12]:

```
%%time
# translation of the pattern into a CTL formula f
f = f"EF({encroachment} & EF(~{encroachment}))"
# split the states whether they satisfy f or not
v = G.split(f)
# split the states whether they are initial or not
v = v.split("INIT")
# print the scenarios satisfying f
v.form(*(v.isin(f) & v.isin("INIT")), variables=controls, normalise="dnf")
```

```
CPU times: user 786 ms, sys: 0 ns, total: 786 ms
Wall time: 785 ms
```

Out[12]:

$\displaystyle \left(BLv \wedge Ig \wedge Ps\right) \vee \left(Ig \wedge Ps \wedge Wl\right) \vee \left(Alt \wedge Ig \wedge Ps \wedge \neg Cb\right)$

If an encroached state is reachable ($\texttt{Ps+} \land \texttt{Ig+}$, see query 1), there are three set of scenarios where at least some encroachment pathways are reversible: (1) with browsing livestock $\texttt{BLv}+$, (2) with wildlife $\texttt{Wl+}$, (3) at high altitude $\texttt{Alt+}$ with crops allowed $\texttt{Cb-}$.

### Invariance pattern : AG(EF(Subsistence))¶

Pattern : $\forall G(\exists F(\texttt{Subsistence}))$

Pattern description : Subsistence states necessarily remain forever reachable.

In [13]:

```
%%time
# translation of the pattern into a CTL formula f
f = f"AG(EF({subsistence}))"
# split the states whether they satisfy f or not
v = G.split(f)
# split the states whether they are initial or not
v = v.split("INIT")
# print the scenarios satisfying f
v.form(*(v.isin(f) & v.isin("INIT")), variables=controls, normalise="dnf")
```

```
CPU times: user 1.52 s, sys: 3.17 ms, total: 1.52 s
Wall time: 1.52 s
```

Out[13]:

$\displaystyle \left(Ps \wedge \neg Ig\right) \vee \left(Alt \wedge Ps \wedge \neg Cb\right) \vee \left(Alt \wedge Wl \wedge \neg Cb\right)$

There are three sets of scenarios where subsistence remains reachable whatever happens : (1) under pastoralism $\texttt{Ps+}$ without intensive grazing $\texttt{Ig-}$, (2) at high altitude $\texttt{Alt+}$ with crops allowed $\texttt{Cb-}$ and with pastoralism $\texttt{Ps+}$ , or (3) at high altitude $\texttt{Alt+}$ with crops allowed $\texttt{Cb-}$ and with wildlife $\texttt{Wl+}$.

### Reachability & Invariance pattern : EF(EG(Subsistence))¶

Pattern : $\exists F(\exists G(\texttt{Subsistence}))$

Pattern description : It is possible to reach a state from which the subsistence can persist forever.

In [14]:

```
%%time
# translation of the pattern into a CTL formula f
f = f"EF(EG({subsistence}))"
# split the states whether they satisfy f or not
v = G.split(f)
# split the states whether they are initial or not
v = v.split("INIT")
# print the scenarios satisfying f
v.form(*(v.isin(f) & v.isin("INIT")), variables=controls, normalise="dnf")
```

```
CPU times: user 1.47 s, sys: 0 ns, total: 1.47 s
Wall time: 1.47 s
```

Out[14]:

$\displaystyle \left(BLv \wedge Ps\right) \vee \left(Ps \wedge \neg Alt\right) \vee \left(Cb \wedge Fb \wedge Ps \wedge \neg Ig\right)$

There are three sets of scenarios where it is possible to reach a state from which subsistence can persist forever: (1) under pastoralism $\texttt{Ps+}$ with browsing livestock $\texttt{BLv+}$, (2) at low altitude $\texttt{Alt-}$ with pastoralism $\texttt{Ps+}$, or (3) with fire banned $\texttt{Fb+}$ as well as crops $\texttt{Cb+}$ and with pastoralism $\texttt{Ps+}$ but without intensive grazing $\texttt{Ig-}$.

## References¶

- (Liao et al. 2018a) Liao C, Clark PE, DeGloria SD. Bush encroachment dynamics and rangeland management implications in southern Ethiopia. Ecology and Evolution. 2018;8(23):11694–11703. doi:10.1002/ece3.4621.
- (Liao et al. 2018b) Liao C, Clark PE. Rangeland vegetation diversity and transition pathways under indigenous pastoralist management regimes in southern Ethiopia. Agriculture, Ecosystems & Environment. 2018;252:105–113. doi:10.1016/j.agee.2017.10.009.
- (Liao thesis, 2016) Liao C. Complexity In The Open Grazing System: Rangeland Ecology, Pastoral Mobility And Ethnobotanical Knowledge In Borana, Ethiopia [PhD Thesis]. Cornell University; 2016. Available from: https://hdl.handle.net/1813/43578.
