# Supplementary material for: Model-checking ecological state-transition graphs
Source: PLoS Comput Biol. 2022 Jun 6;18(6):e1009657. doi: 10.1371/journal.pcbi.1009657 (PMC9203009; doi:10.1371/journal.pcbi.1009657)
Supplement: Attachment — Submitted filename: Response_letter.pdf [file pcbi.1009657.s006.pdf]

April 6 2022

Description of the revision and responses to the review comments
Model-checking ecological state-transition graphs

Dear PLOS Computational Biology editors and reviewers,

Thank you for your enlightening reviews. We revised the manuscript in depth, taking into account the reviewers comments that were highly helpful. We believe that this revision improved the manuscript and helped its positioning. We made several changes in the manuscript, in particular:

1. We directed the manuscript more clearly towards an audience of ecologists handling STGs and interested in analysis tools. We articulated the contributions of the manuscript and its place with respect to existing works. We revised abstract, introduction, surrounding context, discussion and conclusion accordingly.

2. We gave more precise definitions of the mathematical objects presented within the manuscript. Those definitions are given within the main text for core concepts, and within the new "S1 Appendix" for our implementation.

3. We expanded the presentation of the application by detailing the modelling process, the validation of the resulting model and the ecological interpretation of its results. We revised the Materials and methods and Results accordingly, and added a new "S3 Appendix" breaking down how we built the model from the data available in the literature.

4. We revised the discussion according to the new positioning of the manuscript. In particular, we addressed the limitations of model-checking ecological STGs, we discussed the choices made in the application, and we sketched bridges between existing studies in ecology and available model-checking frameworks.

In addition, please find below our detailed responses to the review comments (with the original review comments given in italic).

Sincerely,

Colin Thomas, on behalf of all the authors.

1 Responses to reviewer #1

The submitted manuscript promotes the model checking technique in the area of ecological systems. The article is interesting to read, however some issues are for me not clear. Below I include point-by-point my comments:

1. *Line 77: what is "EDIT STMs database"? It should be revised.*
   The "EDIT STMs database" is a catalogue encompassing various state-and-transition models (STMs) managed by the USDA Natural Resources Conservation Service, the USDA Agricultural Research Service (ARS) Jornada Experimental Range and New Mexico State University (NMSU). While it seems for now limited to the USA, it is aiming to be a global tool helping standardisation and access to STMs. We think that it is both a helpful information to a reader seeking a database of state-transition graphs, and an hint of the interest for ecological state-transition graphs. We revised the sentence by describing more precisely the EDIT database, and added a reference to a publication introducing this database along with its purpose (line 88).

2. *Line 143 Fig. 3: it should be "whether" instead of "wether".*
   Indeed, we modified the figure accordingly.

3. *Line 257: a short technical specification of the computer that has been used should be given (including CPU, RAM, etc.).*
   We added the required specification (lines 318-320).

4. *Line 258 Table 3: how are the control variables embedded? It is not clear from the rest of the article.*
   We added information on controls in several places along the paper (lines 133-134, 154-163, 283-288). We detailed both how the controls are embedded within the model and selected in the results.

5. *Line 261: "Thus the STGs drawn below (. . .)" → where?*
   We removed this paragraph when we rewrote the results section.

6. *In general – section 2: must be extended to include more information. It is now a little bit confusing.*
   We rewrote section 2 (Results), detailing the validation of the model, the methods used to compute the results and the results themselves.

7. *In general – section 4: please point out the contributions of the paper.*
   We added a paragraph at the end of section 4 (lines 500-508) that explicitly points out the contribution.

8. *In general – section 4: what are the limitations of applying model checking to ecological STGs? Appropriate information should be added.*
   We added a discussion about the benefits and limitations of applying model-checking to ecological STGs in section 3.1. Moreover we reminded the main limitation in section 4 (lines 492-493)

2 Responses to reviewer #2

The authors propose an interesting approach of using model-checking for scenario analysis of ecological systems.

A) *General remarks on the positioning of the article:*

   1) *This approach is not new and has already been published in the following articles:*
      C. Largouët, M.-O. Cordier, Y.-M. Bozec, Y. Zhao, G. Fontenelle. Use Of Timed Automata And Model-Checking To Explore Scenarios On Ecosystem Models, Environmental Modelling and Software, Elsevier, 2011
      M.-O. Cordier, C. Largouët, Y. Zhao. Model-Checking an Ecosystem Model for Decision-Aid. IEEE 26th International Conference on Tools with Artificial Intelligence, 2014

      The authors claim that the above mentioned work is only applied in the field of agronomy, which is absolutely not true (since it is indeed about ecosystems as presented here) and that it was used in a "model-checking mindset" (what does that really mean?).
      The above mentioned work was done in collaboration with ecologists (co-authors of the publication) using model-checking, query patterns, scenario testing and applied on real applications with validation of the results. This is exactly the approach that the authors wish to develop here.

      The authors are asked to situate their own approach in the continuity of these previous works, precursors of the domain, in the introduction section, and to place this new work with respect to the state of the art which is not inexistent contrary to what is stated.

The authors are also asked to clearly specify where the novelty of this work lies in relation to what has already been published (which is currently unconvincing in the actual version of this paper).

We agree with reviewer 2 that the first version of the manuscript did not presented the precursory articles as such. Indeed both approaches are very similar: both use model-checking on ecosystem models, with query patterns and scenario testing. We revised the manuscript accordingly to describe our approach in the continuity of these precursory works, both in the introduction (lines 36-37), in the surrounding works (182-185) and in the discussion (lines 475-482). We also tried to clearly state the novelty of our work with respect to the precursors: while the precursory articles are case studies presenting a concrete modelling approach (timed automata) with a validated application, we offer an general overview of both STGs in ecology and model-checking methodology.

2) *The approach proposed by the authors is ambiguous. Several times, they defend themselves from using a "computer science" point of view and exempt themselves from giving any formal definitions. However, several concepts are difficult to grasp without more information and globally the proposed approach appears to be too vague and not enough scientifically sound.*
Moreover, the aspects related to the application are not sufficiently developed in the results part. The application is only inspired by the study of a previous publication, is described as a "proof of concept" and does not propose any validation.
I therefore question the applicative side claimed by this submission.

We revised the manuscript to give more precise definitions of the main mathematical objects, while keeping formalisation understandable to the targeted audience. For example, the definition of STG at the beginning of the materials and methods (lines 48-56), or the definition of if-then rule modelling in S1 Appendix. At the same time, we directed the manuscript more clearly toward an audience of ecologists, both in the introduction and in the discussion. In addition, the application has been detailed, notably its validation and the ecological interpretation of the results.

B) *General remarks on the methodology:*

1) *Choice of the model representation formalism:*
Can you please give a formal definition of the STG formalism? Explain if you have defined yourself this formalism, if so how does it differ from existing approaches and what is its interest? If not, give the author's reference. In the source code, this STG seems to be a simple graph, explain if this is the case? Is it a discrete event system formalism? The definition of events is unclear. The non-determinism (L69) stated does not seem to be correct since the events on the arcs are not the same, then it is not a real case of non-determinism as usually defined.

We added a formal definition of the STG concept (lines 48-56), with references to systems biology from which we borrow this term. A STG is indeed a directed graph, whose edges are possibly labelled. It is a quite general concept, that we did not define ourselves. Computer science names it Kripke structure (when the edges are not labelled) or labelled transition system (otherwise), as noted in Section 1.2 (lines 171-175). We use the name State-Transition Graph (STG) because we believe it eases the understanding of a reader unfamiliar with computer science. We choose the name STG to insist on the notions of state and transition, that distinguish them from other graphs like interaction networks. Also the name STG is extensively used in systems biology and for example in Largouët 2011.

A state-transition graph is a kind of discrete-event system, more precisely a discrete-event system defines how the system evolves inside a STG. We added the term "discrete-event system" in the presentation of the STG concept to ease the understanding (line 69). We added a definition of the term "event" as a state transition (line 66).

We added a definition of the adjective "deterministic" for the STG concept (lines 54-56). Indeed the events of a non-deterministic STG are deterministic (i.e. have a single outcome), but the dynamics of the system is not (a state can be the start of several alternative pathways).

2) *Choice of the passage by the formalism of the rules:*
On the method, why is it necessary to go through a rule-based system if we already have the functionning graph (Figure 2 left) as input?

Figure 2 is only an illustration of the if-then rule modelling on a toy model based on the already available STM (Figure 2 left). The whole *Borana model* is more detailed, it encompasses more scenarios and it is based on the transitions described in the literature and not only on the available STMs. We revised the manuscript to make more clearly the distinction between the toy model of Figure 2 and the *Borana model* (lines 143-149). We also added, at the beginning of section 1.1.3 (lines 117-127), a paragraph on the benefits provided by a model computing the STG.

- *As for the STG model, the formalism of the rule-based system is not given. The article must be self-content without having to refer to another publication. It is not a usual production rule-based system which is disturbing. Otherwise, rule R1 could be triggered directly from state 1 (Gr+, Sh-, Tr-) since the premise Gr+ is consistent and is the only condition for R1 to be invoked. The R2 rule subsumes the R1 rule. How is this handled? From a practical point of view, it seems quite surprising that an expert can state both rule R1 and rule R2 (considering that we don't have the left graph). Please develop all these points to emphasize the interest of using rules in your methodology.*

We added a formal definition of the if-then rule modelling in S1 Appendix. Rule R1 cannot be triggered directly from state (Gr+,Sh-,Tr-) because its consequence is already fulfilled in this state (self-loops are not allowed). We revised the manuscript to make that fact more clear (lines 138-142).

Rule R2 is subsumed in rule R1, but applying R1 or R2 do not produce the same transition in general. For example in Fig. 2, applying R1 to the middle state leads to the top state, while applying R2 to the middle state leads to the bottom state. R1 represents high intensity fire, that fuels on grasses (condition) and burns shrubs and trees (consequence). R2 represents low intensity fire, that fuels on grasses (condition) and burns only shrubs (consequence). We believe that an expert can state both rules without knowing the left graph but with knowledge about the consequences of fire intensity.

- *How are the STG states created from the graph? I don't see how it is possible to create the STG states from these 4 rules without knowing them a priori. The logic of this step must absolutely be explained.*

We added a detailed definition of the graph computation in S1 Appendix. The variables definition specifies one or more initial states, from which the whole set of states is computed by the cascading application of rules. Thus the STG states are computed from the 4 rules and the initial state (top state), without knowing them a priori. We revised the manuscript to make that fact more clear (lines 142-143 and caption of Fig.2).

- *In conclusion on this part, it seems to me that this rule-based system is more a graph description language than a real functioning description language.*

Please explain this aspect more clearly and position yourself in relation to domain-specific languages DSL. Reference: Fowler M, Parsons R. Domain-specific languages. Upper Saddle River, NJ: Addison-Wesley; 2011.

The if-then rules is a modelling language, that could also be used as a graph description language. We exemplified this fact in the revised discussion. We also added a paragraph at the beginning of section 1.1.3 (lines 117-127) that discusses the benefits provided by a modelling language not limited to a graph description language. The if-then rules can be seen as a domain-specific language fitted to model the dynamics of ecosystem, yet if-then

rules is a quite general formalism and does not includes features specific to the modelling of ecosystems.

3) *On model-checking:*
If the initial modeling language is a rule-based system why using model-checking approaches and not SAT ones that are relying on logic? Justify the choice of this approach?
Explain what is a "computation tree" even though you are in a symbolic representation (not much explained except in the implementation part when this choice appears - I suppose - as the real choice of your method).

   There are model-checkers based on SAT solvers, but the SAT problem and the model-checking problem do not deal with the same questions (even if there are reductions from model-checking to SAT). In this article we present the model-checking problem to ecologists, because we believe that many questions about ecological STGs are model-checking questions. To ease the understanding for ecologists, we do not discuss the SAT problem in the manuscript.

   Yet we agree with reviewer 2 that the scenario selection problem presented in the application is close to the SAT problem. SAT may certainly be an interesting trail to follow to solve the scenario selection problem. But the manuscript is focused on the model-checking problem with the scenario selection presented as a special instance of the model-checking problem.

   We revised section 1.2.3 presenting our implementation, we gave specific references about our software toolbox and the corresponding model-checking algorithm. The revision of this section does not speak about symbolic computation trees, which was not necessary anymore.

C) *General remarks on the results:*
This results part is very poor and must absolutely be completed if possible with a real application and at least a validation.
In particular in this part a description of the model is missing. The interest of having a STG is to be able to show a part of the provided model. It is impossible to understand what the model looks like which is very annoying for a paper that proposes to focus on ecological modeling. The "proof of concept" is not sufficient.

- *The part related to the obtained model should be better presented. It would be interesting to have a figure presenting and explaining a part of the obtained model Where do the 1141 states come from (we simply understood that there were 8 variables and 7 controls) and why are the graphs disconnected?*
- *"Being fully symbolic, our implementation is highly scalable and can handle models with millions of states." Did you perform scalability analysis before writing such a statement? There is a need for more scientific support.*
- *Can the answers of model-checking queries be compared with the results obtained by Liao (type of queries, results, response time, etc.)? How can you be sure that the yes/no results are correct? The validation of the approach needs to be investigated.*

We revised the manuscript to expand the presentation of the application (sections 1.1.3, 2 and S3 Appendix), focusing on the modelling process, the validation and the ecological interpretation of the results. We added the requested figure (new Figure 6). We expanded the presentation of the controls, and explained with more details why the subgraphs corresponding to each scenarios are disconnected (lines 152-163, 284-288).

We did not perform a rigourous scalability analysis, but we experimented with models up to a few billion states. We revised the manuscript accordingly.

D) *General remarks on the discussion:*
The discussion, like the paper, but even more in this section, does not know how to position itself towards its readers. It proposes many concepts that are too difficult to grasp for an

audience that is not in the computer science field. For more informed people, it is far too vague and not scientifically sound enough. Please find some examples here that should be explicited :

- *What is management policies defined as controls? And what is it "without control"? This point should have been presented before and defined deeper in the methodology part if considered of paramount importance for the authors.*
- *No, probabilities do not make a system hybrid (and still questionable for time).*
- *"Thus they offer fewer analysis capabilities and fewer software tools exist to support them. In general, the more expressive a particular setting is, the less it can be analyzed (because of computational complexity and tools availability), and some questions even become undecidable (i.e. no algorithm can answer them)." Moderate these assertions or support them with references. Some problems are also undecidable with CTL so the argument is difficult to hold.*

This section should be reworked in a more scientific manner.

We agree with reviewer 2 that the discussion of the first version of the manuscript was too difficult to grasp for ecologists and at the same time not formal enough for computer scientists. We rewrote the discussion according to the targeted audience of the manuscript: ecologists handling STGs and interested in analysis tools.
